# Societal Roles of Nonprofit Organizations: Parsonian Echoes and Luhmannian Reframing of the Organization–Society Interface

**DOI:** 10.1177/08997640241241321

**Published:** 2024-04-11

**Authors:** Florentine Maier, Michael Meyer, Christian Burkart, Berta Terzieva

**Affiliations:** 1 WU Vienna University of Economics and Bussiness, Vienna, Austria; 2 FH Joanneum, Kapfenberg, Austria

**Keywords:** societal roles of NPOs, public roles of NPOs, structural functionalism, social systems theory

## Abstract

Nonprofit organizations (NPOs) have long been recognized as playing vital roles in society. Nevertheless, a coherent understanding of how these roles align with broader social theory, and how to conceptualize the interface between nonprofits and society is still lacking. In pursuit of a solid theoretical foundation, we conducted a systematic literature review encompassing 119 publications spanning from 1959 to 2021 that delve into the societal roles of NPOs. We reason that much of prior research has implicitly adhered to a functionalist perspective akin to that proposed by Talcott Parsons nearly seven decades ago. Our review identifies four overarching societal roles fulfilled by NPOs: service delivery, advocacy, integration, and the development of cultural patterns. Recognizing the limitations of Parsonian functionalism, we advocate for a shift toward a neo-functionalist, systems-theoretical framing to allow for an analysis of societal functions that is more sensitive to the heterogeneity and contradictions pervasive in contemporary society.

## Introduction

Nonprofit organizations (NPOs) fulfill essential roles for society—a basic assumption that many members of the research community have agreed upon. Since the early days of nonprofit research, various taxonomies of societal roles have been proposed (e.g., Frumkin, 2002; Kramer, 1981; Moulton & Eckerd, 2012; Neumayr et al., 2009; Salamon et al., 2000) and scholars have expressed concern about nonprofits’ ability to fulfill these roles (e.g., Eikenberry & Kluver, 2004; Frumkin, 2002). However, in our empirical work aimed at understanding issues related to these roles, we have found that existing taxonomies are not entirely satisfactory. Some taxonomies (e.g., Neumayr et al., 2009) outline a few distinct roles, yet their parsimony comes at the cost of neglecting important aspects, such as civic engagement. Others (e.g., Moulton & Eckerd, 2012) specify a multitude of roles, making it challenging to delineate them when analyzing a specific organization (e.g., the boundaries of an innovation role tend to be ambiguous). Furthermore, research on the societal roles of nonprofits has been mostly unclear about underlying social theory. In the face of increasing social and ecological dysfunctionalities, this lack of conceptual clarity has become a serious shortcoming.

This article presents a literature review and a theoretical investigation that aims to facilitate empirical research on nonprofits’ societal roles by proposing a framework that relates to prior research, builds on a theory of society, and comprehensively yet succinctly captures how NPOs contribute to society. In a world of contested normative understandings of the common good, the notion of organizations’ societal roles is also contentious. Like any scientific concept, the role concept is in a dialectical relationship with theory: clear concepts are a prerequisite for developing strong theories, yet concepts gain clarity from the theory they are embedded in (Suddaby, 2010). It is impossible to understand the societal roles of organizations without a theory of society, and yet, prior research on nonprofits’ societal roles has rarely relied on explicit theory.

To address this shortcoming, we conduct a systematic literature review, critically analyzing previous research diachronically and synchronically, to then highlight explicit theories concerning the organization-society interface and uncover implicit assumptions. Throughout this process, we are guided by the following bipartite research question: *How has the concept of societal roles of NPOs been understood in previous research, and how should it be understood in further research to ensure coherence with contemporary social theory and reality?*

Our literature review shows that the bulk of research on the societal roles of NPOs is implicitly—but rarely in any explicitly theorized manner—functionalist. Functionalism is a sociological perspective that views society as a system of interrelated elements that work together to create social order. It examines how elements contribute to the functioning of the whole. In the mid-20th century, structural functionalism, developed by Talcott Parsons, was one of the dominant perspectives in sociology. Parsons argued that for society to function effectively, society as a whole and its subsystems must fulfill specific requirements: adaptation (to society’s environment), goal attainment (in the sense of striving for and setting collective goals), integration (by harmonizing society’s values and norms), and latent pattern maintenance (to maintain values and norms over time). This set of requirements is known as the AGIL scheme (Parsons, 1960; Sciortino, 2015). Today, notions of structural functionalism reverberate in research on nonprofits’ societal roles, but in an obscured way, because this functionalist tradition has fallen out of favor for its anthropomorphizing and unitarist view of society (Giddens, 1979, p. 211). Structural functionalism reifies society as an organism with needs and organizations as organs for meeting those needs. Such a view does not reflect the heterogeneity and inherent contradictions of contemporary society, where, for example, even universalist frameworks such as the UN Sustainable Development Goals, are fraught with conflicts and trade-offs.

Therefore, to do social complexity justice, we propose a reframing based on the neo-functionalist social systems theory developed by Niklas Luhmann (1988, 1995, 2019). Luhmann was a disciple of Parsons, who dissected Parsons’ theory to develop an alternative view of social order. By offering a systems-theoretical reframing, we hope to preserve the useful elements of previous understandings of nonprofit societal roles while bypassing the shortcomings of Parsonian theory. We propose such a reframing not because we believe that functionalist theories are generally superior but because the notion that an organization fulfills a societal role necessitates a functionalist perspective on the interface between organization and society. Social systems theory is currently the most mature theory for illuminating this interface.

Our analysis aims to promote research on how nonprofits—and organizations in general—can contribute to a viable society. Prevailing sociological and economic theories mostly do not illuminate preconditions for the persistence or even flourishing of a society. They bracket this macro-level question while excelling at analyzing micro- and meso-level issues concerning the experiences and actions of individuals and organizations. Much research on the societal functions of nonprofits has worked around this theoretical gap by assuming a commonsense perspective on the societal functions of organizations. Such a perspective, however, tends to be treacherously simplistic, as it revives notions of structural functionalism. Drawing on newer systems theory, we propose a reframing. Social systems theory is dynamic and allows for a more nuanced and realistic consideration of societal sustainability, more attuned to contradictions pervasive in contemporary society.

Our proposal dovetails with systems-theoretical research that analyzes the contributions of nonprofits to multiple societal function systems: not only to the economy and the political system but also to other systems such as science, art, religion, law, sports, health, education, mass media (Will et al., 2018), and perhaps a newly emerging function system of sustainability (Boulanger, 2018). We complement this research by analyzing contributions to society as a whole.

## Method

**Figure 1.**
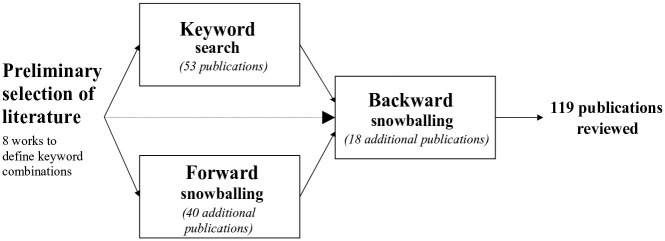
The Systematic Review Process.

To exhaustively delineate the existing taxonomies of nonprofit societal roles, we conducted a systematic literature review following the methodology outlined by Tranfield et al. (2003). We followed four steps (see Figure 1): First, we identified keywords based on a preliminary selection of articles and books that we expected to roughly circumscribe the research field (preliminary selection of literature, see Table 1). Second, we used these keywords to conduct a structured database search. Third, we ran a one-stage forward snowballing search based on the preliminarily selected literature. Finally, using backward snowballing, we included further relevant publications cited in any of the publications identified so far.

**Table 1.** Preliminary Selection of Literature.

Frumkin, P. (2002). *On being nonprofit: A conceptual and policy primer*. Harvard Univ. Press. Kendall, J. (2003). *The Voluntary Sector: Comparative Perspectives in the UK.* London: Routledge. Kramer, R. M. (1981). *Voluntary Agencies in the Welfare State.* Berkeley, Calif.: Univ. of California Press. Moulton, S., & Eckerd, A. (2012). *‘Preserving the Publicness of the Nonprofit Sector: Resources, Roles, and Public Values’.* Nonprofit and Voluntary Sector Quarterly 41 (4): 656–85. Neumayr, M., Meyer, M., Pospíšil, M., Schneider, U., & Malý, I. (2009). *‘The Role of Civil Society Organisations in Different Nonprofit Regimes: Evidence from Austria and the Czech Republic’.* In Civil Society in Comparative Perspective. Emerald Group Publishing Limited. Salamon, L. M., & Helmut, K. A. (1997). *Defining the Nonprofit Sector: A Cross-National Analysis.* Manchester University Press. Salamon, L. M., Hems, L. C., & Chinnock, K. (2000). *The Nonprofit Sector: For What and for Whom?* Vol. 37. Working Papers of the Johns Hopkins Comparative Nonprofit Sector Project,. Baltimore: The Johns Hopkins Center for Civil Society Studies. Salamon, L. M., Sokolowski, S. W., & Regina List. (2004). *Global Civil Society.* Vol. 2. Kumarian Bloomfield, CT.

We made the preliminary selection of literature based on our initial understanding of influential works on the societal roles, functions, or contributions of nonprofits, supplemented by an effort to include geographic variation beyond the United States and a variety of theoretical perspectives.

For the structured keyword search, we scanned the preliminarily selected literature for keywords. Most of these sources used “functions” and “roles” as synonyms (e.g., Kramer, 1981; Neumayr et al., 2009), but some also included “purposes” (e.g., Frumkin, 2002) or “contributions” (e.g., Kendall, 2003). We built search terms by combining these keywords with established synonyms for “nonprofit” (see Table 2, as done by Maier et al., 2016, p. 68). All search terms referring to societal roles were strictly used in their plural form, as the target literature should discuss multiple roles rather than focusing on one. We applied the search terms to the ProQuest database on July 7, 2021, limiting the search to peer-reviewed scholarly articles published in English. The search yielded 1,943 articles as a preliminary result.

**Table 2.** Search Terms for the Structured Keyword Search.

Any of keyword set 1:			
“non*profit roles”	“non*profit functions”	“non*profit purposes”	“non*profit contributions”
“not*for*profit roles”	“not*for*profit functions”	“not*for*profit purposes”	“not*for*profit contributions”
“non*governmental roles”	“non*governmental functions”	“non*governmental purposes”	“non*governmental contributions”
“NPO roles”	“NPO functions”	“NPO purposes”	“NPO contributions”
“NGO roles”	“NGO functions”	“NGO purposes”	“NGO contributions”
“civil societ* roles”	“civil societ* functions”	“civil societ* purposes”	“civil societ* contributions”
“third sector roles”	“third sector functions”	“third sector purposes”	“third sector contributions”
“roles of”	“functions of”	“purposes of”	“contributions of”
**AND**			
Any of keyword set 2:			
“non*profit”			
“not*for*profit”			
“non*governmental”			
“NPO”			
“NGO”			
“civil society”			
“voluntary”			
“third sector”			

To extend our search beyond journal articles and narrow search terms, we searched Google Scholar for publications that referenced the preliminarily selected literature. Due to the high number of results, we limited this search to sources that used at least one of the role synonyms. For each of the eight articles in the preliminary literature, we selected the first 100 results (ordered by relevance).

To screen the results from the database search and snowballing, we employed the following criteria:

1. It is a scholarly publication available in full text and English.
2. It is concerned with the societal roles of NPOs, that is, what nonprofits do for society, regardless of the terminology used (e.g., roles, functions, contributions, and purposes).
3. It discusses specific roles, not just the general claim that nonprofits fulfill roles.
4. It discusses the roles nonprofits fulfill for society at large, not just their roles for individuals or specific fields (e.g., health care).
5. It addresses two or more such roles.

Applying these criteria reduced the number of search results to 93 publications, mainly journal articles and a few books. Two of the authors screened the literature in parallel, with exclusions requiring consensus.

Finally, we read the literature identified so far and, in one backward snowballing step, added relevant publications that had escaped previous search attempts and met the abovementioned criteria. This resulted in 18 additional publications. To enable the reader to assess the appropriateness of our choices, the final list of included literature is available as an online appendix.

The final set of relevant literature was imported into NVivo for coding according to the theories and conceptualizations of societal roles used. The codes were developed inductively. That is, we read the texts, coded relevant passages into categories that emerged from the texts, and successively aggregated these categories to higher levels of abstraction.

## Literature Review

To answer the first part of our research question about how the concept of nonprofits’ societal roles has been understood in previous research, we examine the literature from a diachronic and synchronic perspective.

### Diachronic Analysis

A diachronic review of the literature examines how the understanding of nonprofits’ societal roles has developed over time. Our focus lies on unpacking the theoretical underpinnings that have shaped understandings of societal roles since the beginnings of nonprofit research.

The earliest scholarly work in our sample is a conceptual article by Gordon and Babchuk (1959). Building explicitly on Parsons’ (1951) structural functionalism, the two authors are the first to propose that nonprofits have an expressive and an instrumental function. They argue that nonprofits fulfill instrumental purposes for individuals by offering services and advocating for their interests. These instrumental functions can be characterized as manifest functions, meaning they are intended and widely acknowledged consequences of actions taken by nonprofits. Furthermore, nonprofits serve expressive functions for individuals, such as raising their status. These expressive functions are considered latent functions, that is, unintended and unrecognized consequences of actions.

This seminal distinction between expressive and instrumental function has been widely adopted by nonprofit scholars (see, e.g., Frumkin, 2002; Salamon et al., 2000). Many, however, blurred the notion of expressive and instrumental functions for individuals with expressive and instrumental functions for society (e.g., Frumkin, 2002). As further research adopted the concepts of expressive and instrumental functions, it dropped the surrounding Parsonian framework.

Further seeds of theorizing about nonprofits’ societal roles were planted by Weisbrod (1977) and Hansmann (1980) in debates about the origins of the nonprofit sector. These contributions are rooted in neoclassical economics and, hence, do not explicitly refer to structural functionalism. Still, their line of argument bears the mark of functionalist thinking (Laville & Evers, 2004, p. 21): Both scholars explain the existence of NPOs by accentuating nonprofits’ economic role as service providers. Weisbrod (1977) proposes a theory of government and market failure, suggesting that nonprofits fulfill a role as providers of merit goods and collective goods when markets and governments fail to supply them. In his contract failure theory, Hansmann (1980) adds to this argument and suggests that, in the presence of information asymmetry, consumers trust nonprofits more than businesses because of nonprofits’ non-distribution constraint.

Another seminal contribution is Kramer’s (1981) book, in which he states, in a general reference to the research field, that “[m]ost discussions of the character, goals, and functions of voluntary agencies imply the performance of four organizational roles” (Kramer, 1981, p. 9): vanguards, advocates, value guardians, and service providers. Remarkably, Kramer (1981) distances himself from the notion of function (“a term that has been preempted by several disciplines,” Kramer, 1981, p. 9) and provides a decidedly non-functionalist—but rather symbolic interactionist or cognitive (Biddle, 1986)—definition of the “role” concept: “the pattern of expected organizational behavior relating to the position of the voluntary agency in the social welfare system” (Kramer, 1981, p. 9). Here, the government is the one who expects specific behavior from the NPOs.

In contrast to Kramer (1981), most of the following work took a different path, adhering to a more or less explicitly functionalist view.^1^ For instance, in his influential “conceptual and policy primer,” Frumkin (2002) recognizes four societal functions of nonprofits that are spread along two dimensions: (1) supply vs. demand side, thus linking back to Weisbrod (1977) and Hansmann (1980) for the demand side; and (2) instrumental vs. expressive, adapting Gordon and Babchuk (1959). The four functions (or roles, as he also calls them) are service delivery (demand/instrumental), social entrepreneurship (supply/instrumental), civic and political engagement (demand/expressive), and values and faith (supply/expressive, Frumkin, 2002, p. 25). Frumkin continues the functionalist tradition by linking Weisbrod’s (1977) government/market failure theory with the question of which roles nonprofits perform (Frumkin, 2002, p. 16). In his view, nonprofits’ fulfilling their societal roles is mainly a challenge of satisfying stakeholder expectations to secure the organization’s survival (Frumkin, 2002, p. 163). Although Frumkin did not elaborate on a definition of what societal roles are and how they are embedded in a theory of society, his work proved influential, as he was far-sightedly concerned about imbalances: commercialization, marketization but also ethnic and religious tribalism might threaten nonprofits’ ability to fulfill their societal roles.

Among recent contributions, Moulton and Eckerd (2012) stand out as the latest significant juncture. The authors review the state of research on nonprofits’ societal roles and propose an extensive list of such roles (service, innovation, individual expression, social capital creation, political advocacy, and citizen engagement) and an index for measuring them. Like most previous research, they do not provide a definition of a societal role or integrate such roles into a theory of society.

### Synchronic Analysis

A synchronic analysis considers the literature in a cross-sectional synopsis. Doing so, we find a variety of taxonomies ranging from two (e.g., Fyall, 2017) to ten societal roles (Diamond, 1994). Examining these taxonomies reinforces the impression that this research has ties to structural functionalism for two reasons: First, societal roles and functions are largely viewed as synonymous, which is characteristic of structural functionalism. Second, understandings of roles in the research field overlap with Parsons’(1951) AGIL scheme. Both reasons are discussed below.

#### Roles as Functions

Relatively consistent language is used in research to refer to nonprofits’ societal roles (see Table 3). Most publications (99 out of 119) use the term “role” for the role concept at least once, and 76 publications use “function” at least once. “Contribution” and “purpose” are rarer, with only 31 and 12 publications, respectively, mentioning these terms. Beyond these, we did not find any other major terms.

**Table 3.** Frequency of the “Role” Term and Potential Synonyms.

Term used in previous research	Number of publications using the term
Role	99
Function	76
Contribution	31
Purpose	12

All but one of the reviewed publications use “roles” and “functions” as synonyms; only two publications use “purposes” as the main concept (e.g., Neem, 2006). Moreover, several publications explicitly differentiate between what organizations declare their purpose and what roles they fulfill for society. For example, Warren (2003) argues in his article on “the political role of nonprofits in a democracy” that:

[P]urpose-based schemas do not really tell us what we want to know anyway. We need functional designations. We want to know what the effects of organizations are within the democratic system, not just what purposes they have. Thus, for example, an organization can seek to serve purposes that are not especially public at all, much less democratic: religious organizations, for example, may seek to save their members’ souls. But in so doing, these organizations may teach individuals the habits of association that Tocqueville, for one, saw as essential to the democracy-sustaining habits of citizens. (Warren, 2003, p. 47)

Still, organizations’ statements about their purposes may serve as—admittedly incomplete—indicators of their societal roles (Daniel & Moulton, 2017).

Most publications use the terms “roles” and “functions” interchangeably, while the term “contributions” typically defines roles or functions as equivalent to what nonprofits contribute to society (e.g., Salamon, 2003, p. 11: “Roles and functions: Quite apart from their economic importance, nonprofit organizations make crucial contributions to national and community life”). This is a peculiar understanding, typical for a functionalist perspective (see Laville & Evers, 2004, p. 21), concerned with understanding what something is useful for and which problems it solves.

Research beyond the nonprofit sector rarely refers to organizations as having societal roles. In most social sciences, the role concept refers to the individual level, that is, a person fulfilling a particular social role (e.g., Biddle, 1986). Parsons (1951, p. 16) defines a role as “what the actor does in his relations with others seen in the context of its functional significance for the social system.” Parsons derived his conceptualization of societal functions (i.e., adaptation, goal attainment, integration, and latent pattern maintenance) from group dynamics, specifically from the work of Bales (1950), who clustered the problems encountered by small groups into four functional categories. Parsons found that these categories apply not only to groups but also to society. Parsons and Bales collaborated on several projects, including an analysis of the American family that contributed to Parson’s reputation as a conservative (Parsons & Bales, 1955). In family sociology, Parson intertwined the concepts of functions and roles: In the family, the male gender role is responsible for adaptation and goal attainment, and the female gender role is responsible for integration and the maintenance of latent patterns. Parsons even uses the term “functional role” as a synonym, for example:

[There is the] differentiation between what may be called “instrumental-adaptive” and “expressive-integrative” functional roles, the one being concentrated primarily—though by no means exclusively—on responsibility for the welfare of the system as a system in relation to the exigencies of its situation and the achievement of its goals, the other on the management of the internal motivational tensions of the members and their solidarity with each other to form an integrated group. Though there are many differentiations and qualifications, some of which will be noted later, this is in the broadest respect, the main axis of the differentiation of sex roles in all societies. (Parsons, 1964/1970, p. 43f)

This quote underscores how strongly related the concepts of role and function are in Parsonian theory (although there are differences between these concepts). It also sheds light on why Parsons’ reputation declined in the 1960s and why many younger scholars began to avoid the concept of societal functions.

After this discussion about the functionalist perspective implied in speaking about societal roles or functions, we now turn to an examination of nonprofits’ specific societal roles discussed in research.

#### Four Core Roles

By examining the literature, it becomes evident that the role concepts closely resemble the functions outlined in Parsons’ AGIL scheme. This scheme points to four conditions that any action system (e.g., an organization or a society) must meet if it is to persist (Parsons, 1960; Sciortino, 2015). First, all these systems have to solve the adaptation (A) problem, which stands for their capacity to adapt to the conditions of their environment by acquiring, processing, and distributing scarce material and symbolic resources. Second, any system must set collective goals and mobilize resources to achieve them, that is, fulfill the goal attainment (G) function. Third, any system must adjust and coordinate relationships and interactions among its elements to avoid disruption—an exigency called integration (I). The integration function also includes managing conflicts. Fourth, any system must maintain its latent patterns (L), for example, norms, values, and culture, by generating commitment to shared values and identities.

Most research understands nonprofits to have societal roles that roughly parallel those outlined in the AGIL framework. Table 4 provides an overview of the different roles identified in the literature, along with their frequency of mention. To promote clarity, we have grouped publications that refer to similar or identical roles, even if they use different labels for these roles, under the most bespoke and commonly used role descriptors. These descriptors summarize converging views about nonprofits’ societal roles in previous research. It should be noted that the frequencies alone do not provide a rigorous conceptualization of these roles. However, this overview shows which roles form the consensual core of the nonprofit role concept.

**Table 4.** Frequency of Societal Roles Suggested in Previous Research.

Role	No. of publications suggesting this role
**Service**	**112**
**Advocacy**	**108**
**Integration**	**64**
Community building (i.e., social integration)	43
Systemic integration	37
*Civic engagement (i.e., connecting individuals to social systems)*	*27*
*Convenor (i.e., connecting social systems)*	*18*
**Maintenance and change of cultural patterns**	**62**
Civic education	54
Expressing values	28
**Innovation**	**37**

##### Service

Service provision is the most widely acknowledged societal role of nonprofits. It is named by 112 of the 119 reviewed publications. The vast majority of publications use the terms service provision or service delivery.

This role corresponds to the adaptation function in the AGIL scheme. As a societal function, according to Parsons (1969; also see Sciortino, 2015), adaptation is about providing and allocating the material and symbolic resources to ensure that society is adapted to the conditions of its environment (including the natural environment and individual actors’ mental and physical states). Parsons associated this function with the economy, which specializes in the production and allocation of goods for a variety of goals.

This description resonates with the typical understanding of the service role as shorthand for nonprofits providing goods and services and, hence, often compensating for market failure (Salamon et al., 2000, p. 5). Furthermore, it resonates with Neumayr et al.’s (2009) notion that the service role is oriented toward the economic system because it is about producing outputs that can be priced.

On closer consideration, there are two problems with these attempts to conceptualize the service role of nonprofits. First, using the criterion of whether a nonprofit activity can be priced would either exclude goods that experience market failure, which constitutes a significant portion of nonprofits’ work, or it would encompass almost any activity because whether something could be priced has become a matter of how far one wants to go with neoclassical economic thinking. Second, defining the service role as the provision of goods and services is tautological and fails to differentiate it clearly from other roles. This problem also arises from the extensive reach of neoclassical economic theory, which defines goods as anything—including services—that satisfies human wants (see Bruni, 2005, on Marshall’s seminal definition). Consequently, activities such as advocacy, integration, and the development of cultural patterns could be understood as services. In the section on theoretical reframing, we propose a more precise definition of the service role.

##### Advocacy

Advocacy is the second most widely recognized societal role of nonprofits, mentioned by 108 of the 119 publications. Most previous research uses the term advocacy to refer to this role; synonyms are variants of policy (e.g., policy-making, policy formulation), representation, watchdog, and voice.

This role corresponds to the goal attainment function in the AGIL scheme. According to Parsons (see the summary by Sciortino, 2015), this function is about society attaining its collective goals. Parsons sometimes implied the selection of goals as part of this function (Parsons, 1960), while at other times, he argued that societies have goals due to their values and the situation they find themselves in (Parsons, 1969). Either way, the goal attainment function is about managing concerted action for collective purposes (Sciortino, 2015). Parsons associated this function with the “polity,” which denotes a political realm that includes and goes beyond government institutions. It parallels the economy and is concerned with the generation and allocation of power (Parsons, 1956).

This understanding corresponds to advocacy as a nonprofits’ societal role that comprises efforts to influence public policy (Moulton & Eckerd, 2012). Nonprofits may target governments as well as other powerful actors, such as corporations (Cheah, 2019). Another advocacy tactic is to indirectly influence powerful actors by creating new knowledge, disseminating information, and raising awareness among professionals or the general public (e.g., Shier & Handy, 2015). Jenkins’ (1987, p. 297) classic definition of nonprofit advocacy as “any attempt to influence the decisions of an institutional elite on behalf of a collective interest” captures the spectrum of the advocacy role.

##### Integration

Nonprofits’ integration role is addressed by 64 out of the 119 reviewed publications. Researchers, however, employ various labels, each focusing on a particular aspect of integration. There is widespread awareness of the community-building role of nonprofits, referring to nonprofit activities that build social capital (Putnam, 2000) and foster trust, reciprocity, and inclusion of individuals in communities (43 publications mention this role). Also, the civic engagement role of nonprofits is widely recognized (27 publications). It refers to nonprofits fostering connections between individuals, on the one hand, and organizations, the political system, or society as a whole, on the other. Only 18 publications mention nonprofits’ role as convenors. Convenors connect various organizations or sectors of society, thereby creating a public sphere where exchange can occur. Only recently, scholars have begun to analyze these varied activities under the umbrella concept of integration, differentiating between social integration, when nonprofits connect individuals, and systemic integration, when nonprofits promote civic engagement and act as convenors (Karner et al., 2023).

Community building, civic engagement, and convenorship mirror Parsons’ conceptualization of the integration function. Integration, according to Parsons, involves the adjustment of relationships among members of society, the promotion of unity and cohesiveness, the fostering of pro-social norms, as well as the policing of such norms and the management of conflicts. Parsons attributes this function to what he calls “societal community” (Parsons, 1969, p. 40).

In third-sector research, Van Til (2000, p. xii) drew directly on Parsons and Polanyi to argue that while the economy is responsible for adaptation, politics for goal attainment, and “families, neighborhoods, and communities” for latent pattern maintenance, the nonprofit sector primarily serves an integration role. This, however, remained a minority view, similarly endorsed on a Polanyian basis by Laville and Evers (2004). Most scholars understand integration (or its synonyms) as one of several societal roles of nonprofits.

##### Developing Cultural Patterns

Previous research has widely recognized that nonprofits contribute to reproducing or modifying cultural values, norms, and identities. In the reviewed literature, 62 publications refer to societal roles that correspond to this understanding, particularly civic education (54 publications) and expression of values (28 publications).

The civic education aspect of this role involves instilling values and skills that a good citizen should have in a particular society. Nonprofits may achieve this through intentional educational efforts. Civic education also occurs in unstructured ways when people engage in an NPO and thereby acquire certain skills and values. Research predominantly views civic education as an emancipatory, pro-democratic project. It often refers to nonprofits as “schools of democracy” (e.g., M. Kim, 2017). A few publications point out that in populist or authoritarian regimes, regime-loyal nonprofits push values such as nationalism or religious fundamentalism (e.g., Toepler et al., 2020).

Conversely, value expression is about giving citizens the opportunity to convey their values. A frequent synonym for this aspect, coined by Kramer (1981), is the value guardian role. Many publications frame this exclusively as expressing pro-democratic and pluralistic values. However, Kramer (1981, p. 383) had a broader definition in mind, encompassing the reproduction of “voluntaristic, particularistic, or sectarian values.”

Civic education and value expression align with Parsons’ latent pattern maintenance function, which involves generating commitment to shared values, norms, and identities (Parsons, 1960; Sciortino, 2015). Parsons attributed primacy of pattern maintenance to the “cultural system” (Parsons, 1969, p. 35). To avoid the misconception that this role is solely conservative, we have renamed it to cultural pattern development. Moreover, considering that much of nonprofits’ work on values, norms, and identities is explicit (e.g., preserving ethnic folklore), we follow structural functionalists like Jensen (1976, p. 64) in omitting the qualifier “latent.”

##### Beyond the Four: Innovation

Finally, we want to draw attention to another role of nonprofits occasionally discussed in the literature that, however, may be understood as inherent in the previously mentioned four roles: the innovation (or “vanguard,” Kramer, 1981) role. It is mentioned in 26 of the 109 reviewed publications. Parsons did not conceptualize innovation as a distinct function but understood all four AGIL functions to contribute to both stability and change (Sciortino, 2015). Thus, nonprofits may be innovative in all of their four functional domains.

## Toward a Neo-Functionalist Reframing

Society has become more complex, with more heterogeneity of values and less consensus on common goals than a Parsonian understanding of societal functions assumes. The notion that society could unite around common goals and strive collectively to achieve them, underpinned by social cohesion and shared values, seems misaligned with global and many national realities. As rhetoric, this notion can be found in authoritarian regimes, where unity is maintained at the expense of suppressing dissent. Consequently, research should not be satisfied with a commonsensical and implicitly Parsonian understanding of nonprofits’ roles but needs more robust theoretical underpinnings. We now turn to the second part of our research question, namely, how the societal roles of nonprofits should be conceptualized in future research to ensure coherence with contemporary social theory and reality.

Based on our systematic literature review, we have identified potential for improving theoretical conceptualizations of the societal roles of nonprofits. About half of the reviewed studies did not use explicit theoretical approaches when examining nonprofits’ societal roles. While these studies offer valuable insights into nonprofits’ contributions across different contexts, the absence of theoretical frameworks complicates their synthesis. Our discussions on the service and innovation roles have revealed a need for more clarity regarding the distinctions between specific roles. Moreover, the proliferation of role taxonomies and subcategories further exacerbates the problem and increases the risk of important roles being overlooked in research designs. These factors hinder the ability of the field to consolidate knowledge about what helps or hinders nonprofits in fulfilling various roles in society.

While the nature of societal roles remains theoretically unclear, some studies provide theory-based explanations for why nonprofits focus on specific roles. New institutionalism and resource dependency theory (e.g., M. Kim, 2017), as well as historical institutionalism (e.g., T. Kim, 2008), including social origins theory as a variant (Salamon & Anheier, 1998), are theoretical frameworks commonly used for this purpose. Such studies provide valuable insights, but their explanatory concepts (e.g., institutional isomorphism, resource dependence, power relations among different groups in society) have stronger theoretical underpinnings than their explanandum of societal role fulfillment. Nonprofit roles are assumed to exist without considering how they fit into the main theory used. For example, although new institutionalism provides potential starting points for formulating societal roles of organizations, such as promoting the rationalization of society (Drori et al., 2009), what this means for the theorization of nonprofits’ societal roles remains opaque.

Against this backdrop, we propose a systems-theoretical reframing of the societal role concept that might enable such an integration into a broader theory of society. We see three advantages of such reframing: First, social systems theory allows for easy integration with other theories, as it is a “harmless” (Youssef, 2021) theory: it provides analytically precise descriptions but no causal explanations. A systems-theoretical framing of societal roles could thus be combined with other theories to explain, for example, the prevalence of certain roles in certain organizations. Second, it salvages the consensus on the societal roles of nonprofits in most previous research, with its widespread overlaps with the Parsonian AGIL scheme, but does not reintroduce problematic Parsonian baggage. Importantly, it does not presume a homogeneous society that jointly pursues shared goals. Third, systems theory fills a gap in the social theory landscape. In response to the limitations of structural functionalism, many scholars interested in the positive societal impacts of organizations have shifted from abstract theorizing to more concrete research areas (e.g., social impact measurement). Other scholars interested in abstract questions have shifted from asking how organizations contribute to society to how social context influences organizations. Systems theory, however, is at a highly abstract level interested in what organizations do for society, that is, in the functioning of society as a whole (Luhmann, 2012), and in how macro-level coordination failures threaten this functioning (Luhmann, 1985).

Before suggesting a systems-theoretical reframing of nonprofits’ societal roles, two additional preliminaries are needed (Youssef, 2021). First, systems theory differs from other theories as it hardly postulates relationships between concepts from which empirically testable hypotheses could be directly deducted. Instead, its strength lies in precise descriptive analysis, providing a toolkit of concepts and thinking patterns that can be used to reformulate research problems. As self-critically noted by systems theorists (Youssef, 2021), this entails the risk of merely reformulating established knowledge in a new terminology. Second, systems theory has a unique vocabulary, making it challenging to explain to those unfamiliar with it. One must choose between using simpler language and, thus, risking criticism from theory experts, or alienating theory novices by being incomprehensible. Here, we will opt for the first of these two evils.

In the following, we will outline the concepts of systems theory that are relevant to the analysis of societal roles (resp. functions) of organizations: functional differentiation, society, organizations, and functions.

Modern society is functionally differentiated, that is, there are several distinct function systems such as the economy, the political system, the scientific system, and the health care system. This functional differentiation is not about a division of labor but rather about a multiplication of horizons of meaning (Will et al., 2018). Each function system consists of communications in a distinctive binary code denoting success or failure, for example, the economy communicates in payments/non-payments, the political system in domination/subordination, the scientific system in truths/falsehoods, and religion in transcendence/immanence. Different function systems process the same phenomenon differently: for example, a pandemic may simultaneously be processed as an economic risk, a force of political disruption, a phenomenon in need of scientific explanation, or a warning call by Gaia.

Society, from the perspective of systems theory, is the totality of experiences and actions that are accessible to one another through communication (Luhmann, 1981). Contemporary society is, hence, global. In earlier societies differentiated by social strata rather than function systems, one subsystem had clear interpretive priority over the others. For example, a pandemic might have been understood first and foremost as divine punishment. In the age of functional differentiation, there is one society, but it is described differently from the viewpoint of different function systems. Any event can be economized, politicized, scientized, religionized, and so on. Hence, functional differentiation turns society into functional multiverses of different and incommensurable perspectives (Will et al., 2018). Each function system externalizes all issues that do not pertain to its key concern. Whatever issues fall between the cracks of existing function systems—such as issues of ecological sustainability—have the potential to threaten the continued existence of a functionally differentiated society (Luhmann, 1985).

Organizations are understood as social systems that consist of decisions. Decisions are a certain kind of communication that is normatively expected to involve a justifiable choice between alternatives (Luhmann, 2000). In other words, organizations are collective actors confronted with the generalized expectation that they should be rational. The onus is on these organizations to combine signals from the different function systems, as organizations rely on and contribute to multiple function systems. For example, even highly profit-oriented businesses must deal with the legal system, and current trends toward corporate social responsibility entail that businesses become more multifunctional (Roth et al., 2020). NPOs, in particular, have been characterized as multifunctional (or, from a different theoretical perspective, as “hybrid”). They depend on and contribute to many function systems other than the economy and politics (Will et al., 2018). Nonprofits have also been ascribed responsibility for identifying and critiquing dangerous gaps between function systems (Simsa, 2003).

Based on this understanding of functional differentiation, organizations, and society, we can reframe which functions (or, as a synonym, roles) NPOs fulfill for society. Unlike Parsons, Luhmann (1995) views functions not as per se requirements that certain parts of a system must fulfill to maintain the whole. Instead, Luhmann understands a function as the unity of a “systems problem” and its multiple equivalent solutions, or of a solution and several systems problems equally solved by it (Luhmann, 1995, p. 53). In Parsonian functionalism, functions must be fulfilled to ensure a system’s persistence. According to Luhmann, this is not wrong, but inadequate. Social systems should not be viewed as entities, whose continued existence or disintegration must be explained, but rather as chains of communication whose elements are, in any case, fleeting. The continued existence of a social system means that its communication streams continue.

Today, no more societies end at national borders (Luhmann, 1995). We live in one global society that comprises worldwide communication flows. This society would only end if it dissolved into separate entities with no more communication between them. Taking this as the threshold for successfully fulfilling societal functions would set the bar extremely low. Luhmann, therefore, suggests other criteria for the functioning of a system, such as maintaining the difference between the system and its environment or achieving an appropriate level of complexity.

This complexity criterion seems particularly useful for reframing nonprofits’ societal roles for two reasons: First, compared to other possible criteria, the complexity of a society is highly amenable to empirical operationalization (e.g., using the number of different occupations in a society, Tainter, 1988). Second, all social systems face trade-offs between complexity and sustainability (Tainter, 1988; Valentinov, 2014), and how to handle these trade-offs is arguably the fundamental challenge of contemporary society. Functional differentiation has been the major strategy for how modern societies have increased and simultaneously reduced their overall complexity, though in a paradoxical way. Functional differentiation occurred via the creation of the abovementioned function systems (the economy, politics, the legal system, etc.). Paradoxically, such differentiation of function systems allows for a higher overall complexity by decreasing complexity within each function system. For example, the economy looks only at financial aspects of events, politics at power aspects, and the legal system at legality. Organizations must then piece the signals from the different function systems together to hopefully achieve appropriate levels of complexity within the organization and in society as a whole.

Embedded in such a theoretical understanding of the societal functions of organizations, we believe it is justified to revisit the functions summarized in the AGIL scheme. The scheme remains useful (as Luhmann, 1988, affirmed) as it captures systems problems viewed as relevant by numerous researchers in the nonprofit research field and beyond. We must, however, abandon the Parsonian idea (Sciortino, 2015) that AGIL provides a definitive list of organizations’ societal functions justified by deduction. Instead, we can use the taxonomy of four key roles on an inductive basis as a common ground that many scholars have repeatedly and independently found in their research. The functions of service, advocacy, integration, and cultural pattern development have been pivotal to the nonprofit sector since its early days, and they also encompass recent developments. However, we cannot rule out the possibility that new nonprofit roles may emerge. Moreover, other research fields may develop other formulations of organizational roles, depending on the system problems they address (see Drepper, 2005, from an organization studies perspective).

Against this backdrop, we want to end our proposal with two specific suggestions to remedy ambiguities in the conceptualization of the service and innovation roles. The usefulness of these suggestions will be seen if they can improve instruments for empirically measuring the fulfillment of societal roles.

For the service role, we suggest abandoning attempts to define service provision using concepts from neoclassical economics, which result in either imprecise or overstretched definitions. Instead, we suggest approaching the issue from the tradition of Hirschman (1984): Rather than defining the provision of goods and services as something that can be priced or as a response to market or government failures, focusing on the beneficiaries’ perspective may be more useful. The service role can be understood as the provision of resources used—consumed or invested in—by beneficiaries from a utilitarian perspective. Although one can adopt a utilitarian perspective to invest in and consume relationships built through integration, political effects of advocacy, or cultural patterns, the pursuit of such ends usually occurs from other ethical perspectives. If this remains so, other functions remain distinguishable from the service function.

As for innovation, we propose conceptualizing it not as a distinct role but as inherent in all the other roles. With this proposition, we strive for parsimony on the one hand and want to address two difficulties with operationalizing and measuring a societal innovation role on the other. First, innovation must always refer to something, be it a service, an advocated collective decision, social relations, or a cultural pattern. Second, when it comes to values or solutions to social problems, neither innovation nor conservation can be operationalized clearly in today’s rapidly changing society. The dynamics of contemporary society render the labels “innovative” or “conservative” useless as orientation markers (Luhmann, 1971, p. 398). Conservatives must constantly innovate to preserve the basic features of the existing order (e.g., the stability of the financial system). On the other hand, most so-called progressive ideas are quite old, dating back to the pre-modern era (environmental sustainability), enlightenment (human rights), or at least the social movements of the 1960s and 1970s. There are also nonprofits that work to roll back progress. This creates change, but not of the kind usually called innovation. It, therefore, makes sense to think of innovation, stability, restoration, or regression as possibilities inherent in the other four roles.

## Conclusion

This literature review and theoretical analysis aimed to illuminate how the concept of nonprofits’ societal roles has been understood in previous research. After pointing out common ground and shortcomings in existing research, we offered a proposition on how these roles might be understood in further research.

Our literature review has shown that the debate about nonprofits’ societal roles goes back to the early days of nonprofit research. The idea of nonprofits fulfilling societal roles emerged during the dominance of Parsonian structural functionalism in the social sciences and was at first explicitly linked to this theory (Gordon & Babchuk, 1959). In the 1960s, structural functionalism faced severe criticism and lost its prominence due to its unitarian, anthropomorphizing, and conservative view of society. As a result, the focus in social science shifted away from abstract inquiries into what organizations do for society. Instead, scholars began developing theories like resource dependency theory and new institutionalism to understand how the social environment influences organizations, or they delved into more specific and application-oriented questions about organizations’ social impacts. Research on NPOs, however, continued to be interested in the societal contributions of these organizations, even at an abstract level, as these contributions are a crucial legitimation for nonprofits. However, scholars no longer made explicit references to Parsons and often replaced the term “function” with the less tainted term “role” (e.g., Kramer, 1981). Nevertheless, implicit functionalist thinking continued to shape the field, now coming primarily from neoclassical economics, where the existence of nonprofits was explained through their function to compensate for market and government failures. Further functionalist analyses of nonprofits’ societal roles were put forward (e.g., Frumkin, 2002; Salamon et al., 2000), but without a basis in a theory of society. One exception to this trend has been the microverse of systems-theoretical functionalist analyses, drawing on the work of the sociologist Niklas Luhmann (e.g., Neumayr et al., 2009; Simsa, 2003; Will et al., 2018).

In engaging with the literature in synopsis, we find striking overlaps between the Parsonian AGIL scheme and societal roles of NPOs: service provision as adaptation, advocacy as goal attainment, social and systemic integration, and the development of cultural patterns through civic education and value expression. However, we must reject the structural-functionalist idea that functions can be determined objectively by theoretical deduction, detached from any particular observer’s position. Therefore, Parsons’ claim of providing an exhaustive list of existential problems of social systems must be rejected. Instead, we view the overlaps in research on nonprofits’ societal roles as an indication of plausibility, inductively validating the utility of a taxonomy of four key roles.

The contribution of this analysis is twofold. First, we contribute to research agendas that aim to understand what makes nonprofits focus on particular societal roles (e.g., Karner et al., 2023; M. Kim, 2017; Moulton & Eckerd, 2012; Suykens et al., 2023) and what helps or hinders nonprofits to fulfill those roles well (e.g., Eikenberry & Kluver, 2004; Frumkin, 2002). We have extracted a consensus on nonprofits’ societal roles and suggested how the resulting four main roles can be defined to derive operationalizations and improved measurement tools. The importance of theoretical reframing work lies in its implications for empirical research. Our analysis helps move operationalizations beyond unreflected normativity that assumes that certain roles just must be fulfilled. Systems theory allows for at least two well-reflected approaches to operationalizing role fulfillment: Scholars could establish indicators of role fulfillment based on explicit values such as universal human rights and ecological sustainability. Likewise, they could formulate indicators from the perspective of particular distributions of power, for example, for investigating how certain NPOs contribute to stabilizing authoritarian regimes (Toepler et al., 2020).

Second, we make a theoretical contribution to research that adopts a systems-theoretical perspective to understand the contributions of all kinds of organizations to contemporary society (e.g., Boulanger, 2018; Drepper, 2005; Roth et al., 2020; Valentinov, 2014; Will et al., 2018). Previous analyses from this perspective have mainly focused on organizations’ contributions to various function systems in society and less on contributions to society as a whole. We fill this gap by revisiting Parsons’ AGIL scheme and reformulating it in systems theoretical terms. These systems theoretical analyses are striving to make sense of where our divided and often apparently disoriented society is at and where it is going. With this, we hope to have contributed some fruitful ideas for further exploration in this direction.

Our literature review methodology has limitations to consider when engaging with our findings and suggestions. First, we cannot make claims about the prevalence of functionalist thinking in overall nonprofit research. Our review focused on studies examining nonprofit functions, roles, contributions, or purposes. Given this focus, it was probably inevitable that we would find some functionalist reasoning in the literature. It is difficult to conduct research on societal roles or functions without committing oneself in some way to a functionalist perspective. However, not all nonprofit research is concerned with societal roles, and we do not suggest that nonprofit research is predominantly functionalist. Second, there were limitations to our literature review process. The preliminary selection of literature to determine search terms was based on the authors’ initial understanding of the research field. The keyword-based search of the ProQuest database reduced biases in the preliminarily selected literature but could not cover books and excluded some relevant articles due to imperfect keyword matches. Snowballing allowed for the inclusion of further important books and articles but involved somewhat subjective judgments about relevance. We attempted to mitigate these limitations by working as a team, checking each other’s assessments, and designing the process so that the steps offset each other’s weaknesses. Nevertheless, biases arising from our individual research perspectives cannot be entirely ruled out, and we encourage readers to form their own opinions based on the list of reviewed literature provided in the online appendix.

For further research, we suggest two directions as equally relevant. First, we see a need for fine-tuning, integrating, and consolidating empirical research on the drivers of whether and how NPOs fulfill various societal roles. Theorizing about these drivers could be strengthened by combining a systems theoretical understanding of societal roles with other theories illuminating causal mechanisms, such as resource dependency theory, new institutionalism, or historical institutionalism. The conceptual arguments, qualitative research findings, as well as the still relatively sparse quantitative findings should be examined with more rigorous methods. Further studies might build on the role index developed by Moulton and Eckerd (2012) as well as variants based on it (e.g., Suykens et al., 2023) and further develop this measurement instrument, drawing on the conceptual refinements that we have proposed in this paper.

Second, we see renewed need for a theory of society. We are experiencing disorientation, polarization, and the end of old certainties. In these challenging times, we need to explore new research questions to rethink our understanding of society, the functioning or dysfunctionality of that society, and its relationships with what used to be called the environment and has come back as the more-than-human world that permeates and is permeated by everything that humans do. Research that approaches the societal contributions of nonprofits with those big issues in mind would be insightful because NPOs offer entry points into a wide variety of developments in society. For example: Where is society headed regarding the complexity-sustainability trade-off (Valentinov, 2014)? Are we increasing complexity by developing another differentiated function system of sustainability (Boulanger, 2018), perhaps communicating in the medium of carbon equivalents and based on the distinction between sustainable and unsustainable? Are we heading for a reduction in complexity as parts of our highly differentiated society collapse (e.g., as certain climate risks become uninsurable), and if so, what might a good life look like under such conditions (Thaler, 2024)? Nonprofits are crucial players in struggles over these issues, and analyzing their roles can help us make sense of what is going on.

## Supplemental Material

sj-pdf-1-nvs-10.1177_08997640241241321 – Supplemental material for Societal Roles of Nonprofit Organizations: Parsonian Echoes and Luhmannian Reframing of the Organization–Society Interface Supplemental material, sj-pdf-1-nvs-10.1177_08997640241241321 for Societal Roles of Nonprofit Organizations: Parsonian Echoes and Luhmannian Reframing of the Organization–Society Interface by Florentine Maier, Michael Meyer, Christian Burkart and Berta Terzieva in Nonprofit and Voluntary Sector Quarterly

## Notes

1. A notable exception is Anheier (2005, p. 146), who follows Kramer (1981) and defines the functions of nonprofit organizations as “the normal tasks or roles that nonprofit organizations can be expected to perform.”
