# Supplementary material for: Societal Roles of Nonprofit Organizations: Parsonian Echoes and Luhmannian Reframing of the Organization–Society Interface
Source: Nonprofit Volunt Sect Q. 2024 Apr 11;54(2):327–49. doi: 10.1177/08997640241241321 (PMC11879769; doi:10.1177/08997640241241321)
Supplement: sj-pdf-1-nvs-10.1177_08997640241241321 – Supplemental material for Societal Roles of Nonprofit Organizations: Parsonian Echoes and Luhmannian Reframing of the Organization–Society Interface [file sj-pdf-1-nvs-10.1177_08997640241241321.pdf]

Reviewed Literature

Angley, R. E. (2013). Escaping the Kmara Box: Reframing the Role of Civil Society in Georgia's Rose Revolution. *Studies of Transition States and Societies*, *V*(1), 42-57.

Anheier, H. K. (2009). What Kind of Nonprofit Sector, What Kind of Society?: Comparative Policy Reflections. *American Behavioral Scientist*, *52*(7), 1082-1094. https://doi.org/10.1177/0002764208327676

Berry, J. M. (2005). Nonprofits and civic engagement. *Public Administration Review*, *65*(5), 568-578.

Birgitta Pessi, A., & Grönlund, H. (2012). The Place of the Church: Public Sector or Civil Society? Welfare Provision of the Evangelical Lutheran Church of Finland. *Journal of Church and State*, *54*(3), 353-374. https://doi.org/10.1093/jcs/csr087

Boonpunth, K. C., & Rolls, M. G. (2016). The role of civil society in peacebuilding in southern Thailand. *Journal of Public Affairs*, *16*(4), 376-383. https://doi.org/10.1002/pa.1598

Boris, E., & Mosher-Williams, R. (1998). Nonprofit advocacy organizations: Assessing the definitions, classifications, and data. *Nonprofit and Voluntary Sector Quarterly*, *27*(4), 488-506.

Brainard, L. A., & Siplon, P. D. (2004). Toward nonprofit organization reform in the voluntary spirit: Lessons from the Internet. *Nonprofit and Voluntary Sector Quarterly*, *33*(3), 435-457. https://doi.org/10.1177/0899764004266021

Brinkerhoff, J. M., Smith, S. C., & Teegen, H. (2007). Beyond the "Non": The strategic space for NGOs in development. In *NGOs and the Millennium Development Goals* (pp. 53-80). Springer.

Brodhead, T. (1987). NGOs: In one year, out the other? *World Development*, *15*, 1-6. https://doi.org/10.1016/0305-750X(87)90136-7

Brown, L. D., & Timmer, V. (2006). Civil Society Actors as Catalysts for Transnational Social Learning. *Voluntas: International Journal of Voluntary and Nonprofit Organizations*, *17*(1), 1-16. https://doi.org/10.1007/s11266-005-9002-0

Brown, W. (2017). Classification of Program Activities: How Nonprofits Create Social Value. *Administrative Sciences*, *7*(2). https://doi.org/10.3390/admsci7020012

Cairns, B., Hutchinson, R., & Aiken, M. (2010). 'It's not what we do, it's how we do it': managing the tension between service delivery and advocacy. *Voluntary Sector Review*, *1*(2), 193-207.

Casey, J. (2011, 2011). A new era of collaborative government-nonprofit relations in the US? Nonprofit Policy Forum,

Chandler, S. M., & Johansen, M. (2012). The roles nonprofit organizations play in society in the United States. In *Human Resource Management in the Nonprofit Sector*. Edward Elgar Publishing.

Chaves, M., Stephens, L., & Galaskiewicz, J. (2004). Does government funding suppress nonprofits' political activity? *American sociological review*, *69*(2), 292-316.

Cheah, C. W. (2019). The social-political roles of NGOs: a study on a triadic business network. *Journal of Business & Industrial Marketing*, *34*(5), 994-1004.

Child, C. D., & Grønbjerg, K. A. (2007). Nonprofit advocacy organizations: Their characteristics and activities. *Social science quarterly*, *88*(1), 259-281.

Cumper, G. E. (1986). The changing role of NGOs: no longer the eunuch in the harem? *Health Policy and Planning*, *1*(4), 335-344. https://doi.org/10.1093/heapol/1.4.335

Dana, T. (2015). The Structural Transformation of Palestinian Civil Society: Key Paradigm Shifts. *Middle East Critique*, *24*(2), 191-210. https://doi.org/10.1080/19436149.2015.1017968

Danson, M., & Whittam, G. (2011). Scotland's Civic Society v. England's Big Society? Diverging Roles of the VCS in Public Service Delivery. *Social policy and society*, *10*(3), 353-363. https://doi.org/10.1017/S147474641100008X

De Corte, J., Arys, L., & Roose, R. (2021). Making the Iceberg Visible Again: Service Delivering Experiences as a Lever for NPOs' Advocacy Under a Third-Party Government Regime. *VOLUNTAS: International Journal of Voluntary and Nonprofit Organizations*. https://doi.org/10.1007/s11266-021-00370-6

Dean, J., Goodlad, R., & Rosengard, A. (2000). Citizenship in the New Welfare Market: the Purposes of Housing Advice Services. *Journal of Social Policy*, *29*(2), 229-245. https://doi.org/10.1017/S0047279400005936

Diamond, L. (1994). Rethinking civil society: Toward democratic consolidation. *Journal of democracy*, *5*(3), 4-17.
Diamond, L. (1999). *Developing democracy: Toward consolidation*. JHU press.
Donnelly-Cox, G., Donoghue, F., & Hayes, T. (2001). Conceptualizing the Third Sector in Ireland, North and South. *Voluntas: International Journal of Voluntary and Nonprofit Organizations*, *12*(3), 195-204. https://doi.org/10.1023/A:1012305816257
Donoghue, F. (2009). Public governance roles of third sector organisations in Ireland: A comparison with South Africa. In *Civil Society in Comparative Perspective*. Emerald Group Publishing Limited.
Dubochet, L. (2012). Civil Society In A Middle-Income Country: Evolutions And Challenges In India. *Journal of International Development*, *24*(6), 714-727.
Edwards, B., & Foley, M. W. (2001). Civil Society and Social Capital: A Primer. In B. Edwards, M. W. Foley, & M. Diani (Eds.), *Beyond Tocqueville: Civil Societyand the Social Capital Debate in Comparative Perspective* (pp. 1-16). University Press of New England Series on Civil Society.
Fagan, A. (2005a). Civil society in Bosnia ten years after Dayton. *International Peacekeeping*, *12*(3), 406-419. https://doi.org/10.1080/13533310500074515
Fagan, A. (2005b). Taking stock of civil-society development in post-communist Europe: Evidence from the Czech Republic. *Democratization*, *12*(4), 528-547. https://doi.org/10.1080/13510340500226077
Fernandez, K., & Alexander, J. (2017). The institutional contribution of community based nonprofit organizations to civic health. *Journal of Health and Human Services Administration*, *39*(4), 436-469. http://www.jstor.org/stable/44504691
Flanigan, S. T. (2007). Paying for God's work: A rights-based examination of faith-based NGOs in Romania. *Voluntas: International Journal of Voluntary and Nonprofit Organizations*, *18*(2), 156.
Frumkin, P. (2002). *On being nonprofit: a conceptual and policy primer*. Harvard Univ. Press.
Fung, A. (2003). Associations and Democracy: Between Theories, Hopes, and Realities. *Annual review of sociology*, *29*(1), 515-539.
Fyall, R. (2017). Nonprofits as Advocates and Providers: A Conceptual Framework. *Policy Studies Journal*, *45*(1), 121-143. https://doi.org/10.1111/psj.12165
Göçmen, İ. (2013). The Role of Faith-Based Organizations in Social Welfare Systems: A comparison of France, Germany, Sweden, and the United Kingdom. *Nonprofit and Voluntary Sector Quarterly*, *42*(3), 495-516. https://doi.org/10.1177/0899764013482046
Gordon, C. W., & Babchuk, N. (1959). A Typology of Voluntary Associations. *American sociological review*, *24*(1), 22-29.
Grindheim, J. E., & Selle, P. (1990). The role of voluntary social welfare organisations in Norway: a democratic alternative to a bureaucratic welfare state? *Voluntas: International Journal of Voluntary and Nonprofit Organizations*, *1*(1), 62-76.
Harris, M., Halfpenny, P., & Rochester, C. (2003). A social policy role for faith-based organisations? Lessons from the UK Jewish voluntary sector. *Journal of Social Policy*, *32*(1), 93-112.
He, L. (2010). Social movement tradition and the role of civil society in Japan and South Korea. *East Asia*, *27*(3), 267-287.
Hula, R. C., & Jackson-Elmoore, C. (2001). Governing Nonprofits and Local Political Processes. *Urban Affairs Review*, *36*(3), 324-358. https://doi.org/10.1177/10780870122184885
Ibrahim, U., & Wan-Puteh, S. E. (2018). An overview of civil society organizations' roles in health project sustainability in Bauchi State, Nigeria. *The Pan African medical journal*, *30*, 150-150. https://doi.org/10.11604/pamj.2018.30.150.15851
Ivanova, E., Maier, M., & Meyer, M. (2019). Associations in transition: the business of Russian civil society. *European Journal of International Management*, *13*(5), 709-732. https://doi.org/10.1504/EJIM.2019.101978
Ivanova, E., & Neumayr, M. (2017). The Multi-Functionality of Professional and Business Associations in a Transitional Context: Empirical Evidence from Russia. *Nonprofit Policy Forum*, *8*(1), 45-70. https://doi.org/10.1515/npf-2016-0011
Jezierska, K. (2015). Moral Blueprint or Neoliberal Gobbledygook? Civil Society Frames among Polish Think Tanks. *East European Politics and Societies*, *29*(4), 831-849.

Kamstra, J., & Knippenberg, L. (2014). Promoting democracy in Ghana: exploring the democratic roles of donor-sponsored non-governmental organizations. *Democratization*, *21*(4), 583-609. https://doi.org/10.1080/13510347.2012.751975
Kanti Bandyopadhyay, K., & Khus, T. C. (2013). Changing civil society in Cambodia: in search of relevance. *Development in Practice*, *23*(5-06), 665-677. https://doi.org/10.1080/09614524.2013.800835
Karolewski, I. P. (2006). Civil Society and its Discontents. *Polish Sociological Review*, *154*(2), 167-185.
Keevers, L., Treleaven, L., Sykes, C., & Darcy, M. (2012). Made to measure: Taming practices with results-based accountability. *Organization Studies*, *33*(1), 97-120.
Kendall, J. (2000). The mainstreaming of the third sector into public policy in England in the late 1990s: whys and wherefores. *Policy & Politics*, *28*(4), 541-562.
Kendall, J. (2003). *The voluntary sector: comparative perspectives in the UK*. Routledge.
Kim, J. (2010). A Study of the Roles of NGOs for North Korean Refugees' Human Rights. *Journal of Immigrant & Refugee Studies*, *8*(1), 76-90. https://doi.org/10.1080/15562940903575046
Kim, M. (2017). Characteristics of Civically Engaged Nonprofit Arts Organizations: The Results of a National Survey. *Nonprofit and Voluntary Sector Quarterly*, *46*(1), 175-198.
Kim, T. (2008). The Social Construction of Welfare Control: A Sociological Review on State—Voluntary Sector Links in Korea. *International Sociology*, *23*(6), 819-844.
Kramer, R. M. (1981). *Voluntary agencies in the welfare state*. University of California Press.
Kramer, R. M. (1985). The Future of the Voluntary Agency in a Mixed Economy. *The Journal of Applied Behavioral Science*, *21*(4), 377-391. https://doi.org/10.1177/002188638502100404
Krlev, G., & Lund, A. B. (2020). Social Innovation Ignored: Framing Nonprofit Activities in European News Media. *VOLUNTAS: International Journal of Voluntary and Nonprofit Organizations*, *31*(5), 949-965. https://doi.org/10.1007/s11266-020-00224-7
Kuti, E. (1990). The possible role of the non-profit sector in Hungary. *Voluntas: International Journal of Voluntary and Nonprofit Organizations*, *1*(1), 26-40.
Lagerspetz, M., Rikmann, E., & Ruutsoo, R. (2002). The Structure and Resources of NGOs in Estonia. *Voluntas: International Journal of Voluntary and Nonprofit Organizations*, *13*(1), 73-87. https://doi.org/10.1023/A:1014710229970
Lambie-Mumford, H., & Jarvis, D. (2012). Building better neighbourhoods? Insights into the contributions of local faith-based organisations. *Voluntary Sector Review*, *3*(3), 399-405.
Laville, J.-L., & Evers, A. (2004). Defining the third sector in Europe. In A. Evers & J.-L. Laville (Eds.), *The third sector in Europe* (pp. 11-44). Elgar.
Law, C. K., & Hasenfeld, Y. (1989). The relationships between the public and the voluntary sectors: The case of refugee resettlement services. *Administration in Social Work*, *13*(2), 15-28.
LeRoux, K. (2007). Nonprofits as civic intermediaries: The role of community-based organizations in promoting political participation. *Urban Affairs Review*, *42*(3), 410-422.
LeRoux, K. (2009). The effects of descriptive representation on nonprofits' civic intermediary roles: A test of the "racial mismatch" hypothesis in the social services sector. *Nonprofit and Voluntary Sector Quarterly*, *38*(5), 741-760.
Levine Daniel, J., & Fyall, R. (2019). The Intersection of Nonprofit Roles and Public Policy Implementation. *Public performance & management review*, *42*(6), 1351-1371. https://doi.org/10.1080/15309576.2019.1601114
Levine Daniel, J., & Moulton, S. (2017). Beyond Cans and Capacity. *Nonprofit Management and Leadership*, *28*(1), 47-64.
Lewis, J. (2005). New Labour's approach to the voluntary sector: independence and the meaning of partnership. *Social policy and society*, *4*(2), 121-131.
Lister, S., & Nyamugasira, W. (2003). Design Contradictions in the 'New Architecture of Aid'? Reflections from Uganda on the Roles of Civil Society Organisations. *Development Policy Review*, *21*(1), 93-106. https://doi.org/10.1111/1467-7679.00200
Malhotra, K. (2000). NGOs without aid: Beyond the global soup kitchen. *Third World Quarterly*, *21*(4), 655-668. https://doi.org/10.1080/713701062

Mariani, L., & Cavenago, D. (2013). Redesigning welfare services for policies effectiveness: The non-profit organizations (NPOs) perspective. *Public Management Review*, *15*(7), 1011-1039.
Markström, U., & Karlsson, M. (2013). Towards Hybridization: The Roles of Swedish Non-Profit Organizations Within Mental Health. *Voluntas*, *24*(4), 917-934. https://doi.org/10.1007/s11266-012-9287-8
Marwell, N. P. (2004). Privatizing the Welfare State: Nonprofit Community-Based Organizations as Political Actors. *American sociological review*, *69*(2), 265-291. https://doi.org/10.1177/000312240406900206
Meyer, M., & Leitner, J. (2018). Slack and innovation: The role of human resources in nonprofits. *Nonprofit Management and Leadership*, *29*(2), 181-201. https://doi.org/10.1002/nml.21316
Minkoff, D. C. (2002). The emergence of hybrid organizational forms: Combining identity-based service provision and political action. *Nonprofit and Voluntary Sector Quarterly*, *31*(3), 377-401.
Momen, M. N., Baikady, R., Sheng-Li, C., & Basavaraj, M. (2020). Introduction: Civil Society in the Era of Globalization—Emerging Concerns as a Social Welfare Provider. In M. N. Momen, R. Baikady, C. Sheng Li, & M. Basavaraj (Eds.), *Building Sustainable Communities: Civil Society Response in South Asia* (pp. 1-14). Springer Singapore. https://doi.org/10.1007/978-981-15-2393-9_1
Moulton, S., & Eckerd, A. (2012). Preserving the publicness of the nonprofit sector: Resources, roles, and public values. *Nonprofit and Voluntary Sector Quarterly*, *41*(4), 656-685.
Mukute, M., & Taylor, J. (2013). Struggles for systems that nourish: southern Africa civil society contributions and challenges to the creation of flourishing societies. *Development in Practice*, *23*(5-06), 609-616. https://doi.org/10.1080/09614524.2013.800840
Neem, J. N. (2006). Squaring the Circle: The Multiple Purposes of Civil Society in Tocqueville's Democracy in America. *The Tocqueville Review*, *27*(1), 99-121. https://doi.org/10.3138/ttr.27.1.99
Nesbit, R. (2017). Advocacy Recruits: Demographic Predictors of Volunteering for Advocacy-Related Organizations. *VOLUNTAS: International Journal of Voluntary and Nonprofit Organizations*, *28*(3), 958-987. https://doi.org/10.1007/s11266-017-9855-z
Neumayr, M., & Meyer, M. (2010). *In search of civicness: an empirical investigation of service delivery, public advocacy, and community building by civil society organizations*. Nomos Verlagsgesellschaft mbH & Co. KG.
Neumayr, M., Meyer, M., Pospíšil, M., Schneider, U., & Malý, I. (2009). The role of civil society organisations in different nonprofit regimes: evidence from Austria and the Czech Republic. In *Civil society in comparative perspective*. Emerald Group Publishing Limited.
Neumayr, M., Schneider, U., & Meyer, M. (2015). Public Funding and Its Impact on Nonprofit Advocacy. *Nonprofit and Voluntary Sector Quarterly*, *44*(2), 297-318. https://doi.org/10.1177/0899764013513350
Never, B. (2010). Framing third-sector contributions to service provision: The case of the holy cross dispute. *Nonprofit and Voluntary Sector Quarterly*, *39*(3), 460-477.
O'Connell, B. (1989). What Voluntary Activity Can and Cannot Do for America. *Public Administration Review*, *48*(5), 486-491.
Payton, R. L., & Moody, M. P. (2008). *Understanding philanthropy: Its meaning and mission*. Indiana University Press.
Pestoff, V., Osborne, S. P., & Brandsen, T. (2006). Patterns of co-production in public services: Some concluding thoughts. *Public Management Review*, *8*(4), 591-595.
Pronovost, G. (1998). Voluntary Organizations. *Current Sociology*, *46*(3), 64-70.
Pudjiastuti, T. N. (2003). The Changing Roles of NGOs in Relation to Female Indonesian Labor Migration. *Asian and Pacific Migration Journal*, *12*(1-2), 189-207. https://doi.org/10.1177/011719680301200108
Reuter, M., Wijkström, F., & Meyer, M. (2014). Who Calls the Shots? The Real Normative Power of Civil Society. In M. Freise & T. Hallmann (Eds.), *Modernizing Democracy: Associations and Associating in the 21st Century* (pp. 71-82). Springer New York. https://doi.org/10.1007/978-1-4939-0485-3_6

Salamon, L. M. (2003). *The resilient sector: The state of nonprofit America*. Brookings Institution Press.
Salamon, L. M., & Anheier, H. K. (1997). *Defining the nonprofit sector: A cross-national analysis*. Manchester University Press.
Salamon, L. M., & Anheier, H. K. (1998). Social origins of civil society: Explaining the nonprofit sector cross-nationally. *Voluntas*, *9*(3), 213-248.
Salamon, L. M., Hems, L. C., & Chinnock, K. (2000). *The nonprofit sector: for what and for whom?* (Vol. 37). The Johns Hopkins Center for Civil Society Studies.
Salamon, L. M., Sokolowski, S. W., & List, R. (2004). *Global civil society* (Vol. 2). Kumarian Bloomfield, CT.
Schmid, H. (2004). The role of nonprofit human service organizations in providing social services: A prefatory essay. *Administration in Social Work*, *28*(3-4), 1-21.
Schmid, H., Bar, M., & Nirel, R. (2008). Advocacy Activities in Nonprofit Human Service Organizations: Implications for Policy. *Nonprofit and Voluntary Sector Quarterly*, *37*(4), 581-602. https://doi.org/10.1177/0899764007312666
Shier, M. L., & Handy, F. (2015). From Advocacy to Social Innovation: A Typology of Social Change Efforts by Nonprofits. *Voluntas*, *26*(6), 2581-2603.
Smith, S. R. (2010). Nonprofit organizations and government: Implications for policy and practice. *Journal of Policy Analysis and Management*, *29*(3), 621-625.
Suarez, D., & Hwang, H. (2020). Collaborations and Networks. In H. K. Anheier & S. Toepler (Eds.), *The Routledge Companion to Nonprofit Management* (pp. 317-334).
Sykes, R. E. (1969). An Appraisal of the Theory of Functional-Structural Differentiation of Religious Collectivities. *Journal for the Scientific Study of Religion*, *8*(2), 289-299.
Szarka, J. (2013). From Climate Advocacy to Public Engagement: An Exploration of the Roles of Environmental Non-Governmental Organisations. *Climate*, *1*(1). https://doi.org/10.3390/cli1010012
Tanaka, M. (2011). The Changing Roles of NGOs in Nepal: Promoting Emerging Rights-Holder Organizations for Inclusive Aid. *Voluntas: International Journal of Voluntary and Nonprofit Organizations*, *22*(3), 494-517. http://www.jstor.org/stable/41307820
Tang, S.-Y., & Zhan, X. (2008). Civic Environmental NGOs, Civil Society, and Democratisation in China. *The Journal of Development Studies*, *44*(3), 425-448. https://doi.org/10.1080/00220380701848541
Toepler, S. (2013). Shifting cultural policy landscapes in the USA: What role for philanthropic foundations? *Cultural Trends*, *22*(3-4), 167-179.
Toepler, S., & Anheier, H. K. (2004). Organizational theory and nonprofit management: an overview. *Future of civil society*, 253-270.
Ui, S., Heng, L., Yatsuya, H., Kawaguichi, L., Akashi, H., & Aoyana, A. (2010). Strengthening community participation at health centers in rural Cambodia: role of local non-governmental organizations (NGOs). *Critical Public Health*, *20*(1), 97-115. https://doi.org/10.1080/09581590902829173
Uphoff, N., & Krishna, A. (2004). Civil society and public sector institutions: more than a zero-sum relationship. *Public Administration and Development*, *24*(4), 357-372. https://doi.org/10.1002/pad.313
van der Borgh, C., & Terwindt, C. (2012). Shrinking operational space of NGOs – a framework of analysis. *Development in Practice*, *22*(8), 1065-1081. https://doi.org/10.1080/09614524.2012.714745
Van Til, J. (2000). *Growing civil society: from nonprofit sector to third space*. Indiana University Press.
Wandel, J., & Valentinov, V. (2014). The nonprofit catallaxy: An Austrian economics perspective on the nonprofit sector. *Voluntas: International Journal of Voluntary and Nonprofit Organizations*, *25*, 138-149.
Warren, M. E. (2001). *Democracy and association*. Princeton University Press.
Warren, M. E. (2003). The political role of nonprofits in a democracy. *Society*, *40*(4), 46-51.

Warshawsky, D. N. (2014). Civil society and urban food insecurity: analyzing the roles of local food organizations in Johannesburg. *Urban Geography*, *35*(1), 109-132. https://doi.org/10.1080/02723638.2013.860753

Williamson, R. T., & Rodd, J. (2016). Civil society advocacy in Nigeria: promoting democratic norms or donor demands? *BMC International Health and Human Rights*, *16*(1), 19. https://doi.org/10.1186/s12914-016-0093-z

Woodward, B. (1992). Eastern Europe. *Peace Review*, *4*(4), 18-23. https://doi.org/10.1080/10402659208425672

Yan, X., Lin, H., & Clarke, A. (2018). Cross-Sector Social Partnerships for Social Change: The Roles of Non-Governmental Organizations. *Sustainability*, *10*(2). https://doi.org/10.3390/su10020558

Yu, J., Shen, Y., & Li, Y. (2021). Understanding the Effect of Central Government Funding on the Service and Advocacy Roles of Nonprofit Organizations in China: A Cross-Regional Comparison. *Nonprofit and Voluntary Sector Quarterly*, *50*(1), 186-212.

Zhan, X., & Tang, S.-Y. (2016). Understanding the Implications of Government Ties for Nonprofit Operations and Functions. *Public Administration Review*, *76*(4), 589-600. https://doi.org/10.1111/puar.12515

Zimmer, A., & Freise, M. (2008). Bringing society back in: Civil society, social capital, and third sector. *Civil society and governance in Europe*, 19-45.
